# Asphalt Binder “Skincare”? Aging Evaluation of an Asphalt Binder Modified by Nano-TiO_2_

**DOI:** 10.3390/nano12101678

**Published:** 2022-05-14

**Authors:** Orlando Lima, Cátia Afonso, Iran Rocha Segundo, Salmon Landi, Natália C. Homem, Elisabete Freitas, Amanda Alcantara, Verônica Castelo Branco, Sandra Soares, Jorge Soares, Vasco Teixeira, Joaquim Carneiro

**Affiliations:** 1 Department of Civil Engineering, Institute for Sustainability and Innovation in Structural Engineering (ISISE), University of Minho, 4800-058 Guimarães, Portugal; orlandojunior.jr@hotmail.com (O.L.J.); efreitas@civil.uminho.pt (E.F.); 2 Centre of Physics of Minho and Porto Universities (CF-UM-UP), Azurém Campus, University of Minho, 4800-058 Guimarães, Portugal; catiaj_afonso@hotmail.com; 3 Federal Institute Goiano, Rio Verde 75901-970, Brazil; salmon.landi@ifgoiano.edu.br; 4 Digital Transformation CoLab (DTx), Building 1, Campus of Azurém, University of Minho, 4800-058 Guimarães, Portugal; natalia.homem@dtx-colab.pt; 5 Departamento de Engenharia de Transportes, Universidade Federal do Ceará, Fortaleza 60455-760, Brazil; amanda.a@det.ufc.br (A.A.); veronica@det.ufc.br (V.C.B.); sas@ufc.br (S.S.); jsoares@det.ufc.br (J.S.)

**Keywords:** anti-aging, asphalt binders, nano-TiO_2_, nanomodification, semiconductor nanoparticles, FTIR

## Abstract

Aging by oxidation of asphalt roadway material promotes changes in its physical, chemical, and rheological properties, affecting its hardening and accelerating the degradation of its corresponding asphalt mixture. Titanium dioxide (TiO_2_) has been applied in engineering investigations to promote anti-aging and photocatalytic properties. In this study, a commercial binder was modified with nano-TiO_2_ (using contents of 0.1, 0.25, 0.5, 1, 2, 3, and 6%). It was evaluated by physicochemical and rheological tests (penetration, softening point, mass loss, dynamic viscosity, rheology, and Fourier transform infrared spectroscopy—FTIR) before and after aging by rolling thin-film oven test (RTFOT) and pressure aging vessel (PAV). The results indicated that incorporating nano-TiO_2_ mitigates binder aging, pointing out 0.25% as an optimum modification content for the investigated asphalt binder.

## 1. Introduction and Literature Review

The high degree of exposure in the coupling environment of traffic load, heat, moisture, ultraviolet, oxygen, and others are key factors that contribute to asphalt binder aging [1,2]. Aging by oxidation of the asphalt binder used in roadway surface courses has irreversible effects, such as changing its physical, chemical, and rheological properties—for example, hardening—which accelerates its degradation, resulting in the appearance of pathologies, such as cracking. Aging involves a physical–chemical process that generally develops in two phases: short- and long-term. The former occurs during the manufacture and compaction of asphalt mixtures, mainly by being subjected to high temperatures. The latter occurs during the pavement’s service life and is caused by weathering and can be classified as thermo-oxidative aging and photo-oxidative aging [3,4,5].

The oxidation and polymerization reactions of organic molecules during the service life occur continuously, namely organic product aging [6,7]. UV aging takes place on the asphalt binder by oxidizing saturated and aromatic components into asphaltenes. With the volatilization of lightweight components during the aging process, the asphalt binder becomes harder and brittle rapidly [8]. The increasing brittleness of an asphalt binder can reduce its tensile strength and fatigue resistance, becoming prone to cracks under low-temperature or repeated loading conditions [9,10,11]. The aging process also reduces the adhesion performance of the asphalt mixtures [12].

Road builders and researchers have undertaken efforts to extend asphalt binder durability against climatic conditions and traffic loads, leading them to explore different ways of changing the asphalt composition and improving the binder performance. An outcome is a solid advance in the modification of asphalt binders with dissimilar polymers. More recently, different nanomaterials have been used to enhance the behavior of asphalt binders and mixtures and their mechanical properties. For example, nano-TiO_2_ and nano-SiO_2_ are pointed out as two of the most effective nanomaterials for modifying asphalt binders [13].

The application of certain semiconductors in asphalt road materials can contribute to the reduction in the effects that have undesirable economic and social consequences by (i) promoting the photocatalytic capacity to degrade pollutant compounds [14,15,16,17,18] and (ii) developing an anti-aging capacity to delay the occurrence of pathologies related to the oxidation of bituminous materials [19,20,21].

Concerning the photocatalytic capacity, semiconductor materials, such as zinc oxide (ZnO), titanium dioxide (TiO_2_), and cerium dioxide (CeO_2_), participate in oxidation-reduction (redox) reactions that promote the photodegradation of pollutants. This reaction is initiated by irradiating ultraviolet (UV) light on the semiconductors, which absorb energy equal to or greater than their bandgap, promoting an electron from the valence band to the conduction band, generating an electron-hole pair (e^−^/h^+^). The electron-hole pair reacts with water molecules from environmental humidity, forming highly reactive radicals capable of degrading organic pollutants and pollutant gases, such as NO_x_ and SO_2_ [22,23,24].

Regarding aging, the literature reports some works involving materials to promote anti-aging capacity, namely asphalt binders. Rocha Segundo et al. concluded that it was possible to obtain softer asphalt binders and better results of short-term aging resistance by using at least 0.08% TiO_2_ [19]. Chen et al. found that asphalt binders modified by 1% vermiculite clay combined with 3% nano-TiO_2_ or with 1% of the same clay and nano-ZnO improve the resistance to oxidation by temperature and UV radiation when compared with the control binder [21]. The Nejad et al. study attests that incorporating 2 to 6% nano-TiO_2_ or nano-ZnO increases the softening point up to 11% and decreases penetration up to 29%. Increasing the percentage of nanoparticles leads to an increase in the viscosity of the asphalt binder [25].

Ren et al. evaluated the modifying effect of nanoparticles on bio-asphalt binders, i.e., nano-SiO_2_, nano-TiO_2_, nano-CaCO_3_, nano-Fe_2_O_3_, and nano-ZnO. The high-temperature performance and aging resistance were improved at increased nanoparticle contents, especially for nano-SiO_2_, while their low-temperature performance was slightly weakened. The effects of the nanoparticles on the functional performance and water stability were insignificant [26].

Zhang et al. analyzed the influence of TiO_2_/CaCO_3_ nanoparticles on the bitumen conventional performance parameters. Regarding the mechanical performance, with the nano-TiO_2_/CaCO_3_ dosage increased, the penetration and ductility of the nano-TiO_2_/CaCO_3_-modified bitumen decreased, while the softening point increased. The rotational viscosity test showed an increased viscosity and reduced bituminous sensitivity by adding nano-TiO_2_/CaCO_3_ [27].

The rheological properties of the asphalt binder were significantly improved by adding TiO_2_ nanoparticles. According to the Ma et al. study, adding nano-TiO_2_ enhanced the high-temperature anti-rutting capacity of the asphalt binder, making the rutting factor vary from 5% to 13% when the temperature range is 40 to 80 °C. In addition, it was also attested that the nano-TiO_2_-modified asphalt mixture has a positive effect on the photocatalytic degradation of CH and NO_x_, especially regarding NO_x_ degradation [28].

Shafabakhsh et al. investigated the presence of nano-SiO_2_ and nano-TiO_2_, considering different percentages of these nanomaterials, and concluded that they improved the asphalt binder properties concerning the rheological behavior and resistance against aging and the asphalt mixtures regarding such damage as rutting and fatigue. The use of 1.2% nano-SiO_2_ and 0.9% nano-TiO_2_ at 40 °C had the best performance, increasing the rutting resistance of the mixture by approximately 100% and the fatigue life of bitumen by 50% [13].

Wu et al. studied the rheological properties and the dynamic and static viscoelastic characteristics of base and nano-TiO_2_/CaCO_3_-modified bitumen. The incorporation of nano-TiO_2_/CaCO_3_ reduced the temperature sensitivity of bitumen (according to the master curve of the complex shear modulus), enhanced the high-temperature anti-rutting, and slightly improved the low-temperature anti-cracking of the bituminous mixture [29].

Yang et al. studied the application of nano-TiO_2_ modified by grafting with PS and rGO to synthesize a TiO_2_/PS-rGO composite and used it as an anti-UV aging agent to prepare a TiO_2_/PS-rGO/styrene–butadiene–styrene (SBS)-modified asphalt binder. The results indicated that the TiO_2_/PS-rGO exhibited excellent UV absorption; the modified asphalt binder TiO_2_/PS-rGO/SBS showed a much better anti-UV aging performance and increased storage modulus and loss modulus. Therefore, the modified asphalt binder improved the elasticity and recovery rate and reduced the creep resistance and high-temperature rutting resistance if compared to pristine SBS-modified asphalt binder [30].

Li et al. analyzed changes in asphalt properties caused by adding the nanomaterial graphene in A-70 asphalt, comparing them after a rolling thin-film oven test (RTFOT). The results revealed that incorporating graphene into an asphalt binder can improve the anti-aging properties. The increase in the softening point (SP) of the base asphalt binder, for example, is 9 °C, while it is only 4 °C for the graphene-modified asphalt binder for a concentration of 1% in weight; the viscosity ratio of the graphene-modified asphalt binder is essentially the same as for the base asphalt. Moreover, the Fourier transform infrared spectroscopy (FTIR) results showed no chemical reactions between graphene and asphalt binder, only physical blending, and the S=O stretching vibration reveals the improved anti-aging properties of graphene-modified asphalt binder [31].

Xie et al. studied the improvement of SBS-modified asphalt binder regarding resistance to ultraviolet (UV) aging and low-temperature performance using nano-ZnO and nano-TiO_2_. The results indicate that these two types of nanoparticles present better compatibility with asphalt binder after surface modification with silane coupling agent (KH-560) and can improve the binding ability between SBS and base asphalt. The nano-ZnO showed a significant effect on the low- and high-temperature performance of the nano-ZnO/nano-TiO_2_/SBS-composite-modified asphalt binder, and nano-TiO_2_ showed a significant effect on the high-temperature performance. In addition, nano-TiO_2_ has a good absorption effect at a wavelength of 365 nm (ultraviolet light), while nano-ZnO is prone to photolysis, and its activity decreases at this wavelength [32].

Other studies also indicated that, when nano-ZnO particles are incorporated into asphalt binder, the viscosity aging index and the mass loss of the modified asphalt binder were inferior to those of the unmodified one after UV aging conditions [33,34,35]. Moreover, the introduction of nano-ZnO particles and expanded vermiculite in the asphalt binder improved its resistance to thermo-oxidative and UV aging at the same time, reducing the deterioration rates of the modified asphalt binder [11,26,32,36].

Liao et al. used nano-TiO_2_ and montmorillonite (MMT) for composite modification of butadiene–styrene-rubber (SBR)-modified asphalt binder and concluded that the effect of nano-TiO_2_ and nano-MMT can delay the process of converting light components to asphaltenes during asphalt binder aging, reducing and almost stabilizing the CMI index of SBR-modified asphalt binder at about 2, decreasing the average stiffness modulus rate from 0.27 to 0.13, and dropping the carbonyl index from 5.16 to 0.28 [37].

Trujillo-Valladolid evaluated the influence of aging on the chemical and mechanical properties of photocatalytic asphalts modified with different percentages of nano-TiO_2_, 3, 5, and 7% by weight, and conventional asphalt binder, PG 64–22. They were exposed to environmental conditions for 6, 12, and 18 months. The results showed that the presence of nano-TiO_2_ increases the resistance to aging and turns the asphalt binder chemically more stable than conventional binders, improving the elastic recovery and the rutting resistance. The photocatalytic efficiency was not degraded by the aging effect. The addition of 10% TiO_2_ also did not represent significant changes in the photocatalytic efficiency, so the 7% TiO_2_ composite is the recommended one [38].

In summary, previous research has concluded that asphalt binders containing nanomaterials, usually nano-ZnO, nano-TiO_2_, and nano-SiO_2_, showed good anti-UV properties according to the UV absorbance results and the physical, rheological, chemical, and morphology aging indexes. Zhang et al. showed that the absorbance results of this nanomaterial are, respectively, nano-ZnO > nano-TiO_2_ > nano-SiO_2_. In addition, the asphalt binders modified with these same three nanomaterials for the concentration of around 2% presented the same rank regarding anti-UV aging properties assumed by their UV absorbance results [39].

Nanomaterials have a great capacity to modify the microstructures of an asphalt binder, changing its performance [11]. Once nanomaterials have a high specific surface area, the use of high percentages can lead to increased stiffness, while low percentages do not. Therefore, it is relevant to study low and high percentages of nanomaterials. Moreover, there is still no consensus or great experience on the use of nanomaterials as asphalt binder modifiers, which requires better evaluation of the use of different percentages and the effects of the amounts of these nanomaterials on the anti-aging properties.

As the previous studies show, different nanomaterials have been used to improve the behavior of bitumen and asphalt mixtures. Thus, this research is devoted to evaluating the anti-aging properties of an asphalt binder modified with a TiO_2_ semiconductor on a nanometer scale. The asphalt binder was modified by the semiconductor in different percentages, and its physical and rheological properties were evaluated to identify the optimal values.

## 2. Materials and Methods

The materials used in this research were the commercial asphalt binder Elaster modified by SBS and the semiconductor TiO_2_ on a nanometer scale. The nano-TiO_2_ semiconductor material was supplied by Quimidroga (Barcelona, Spain), with the following main properties: 80% anatase and 20% rutile crystalline phases, purity > 99.5%, and particle size about 23 to 28 nm. The same materials were used in previous research works [14,15,18,19,40].

**Figure 1:**
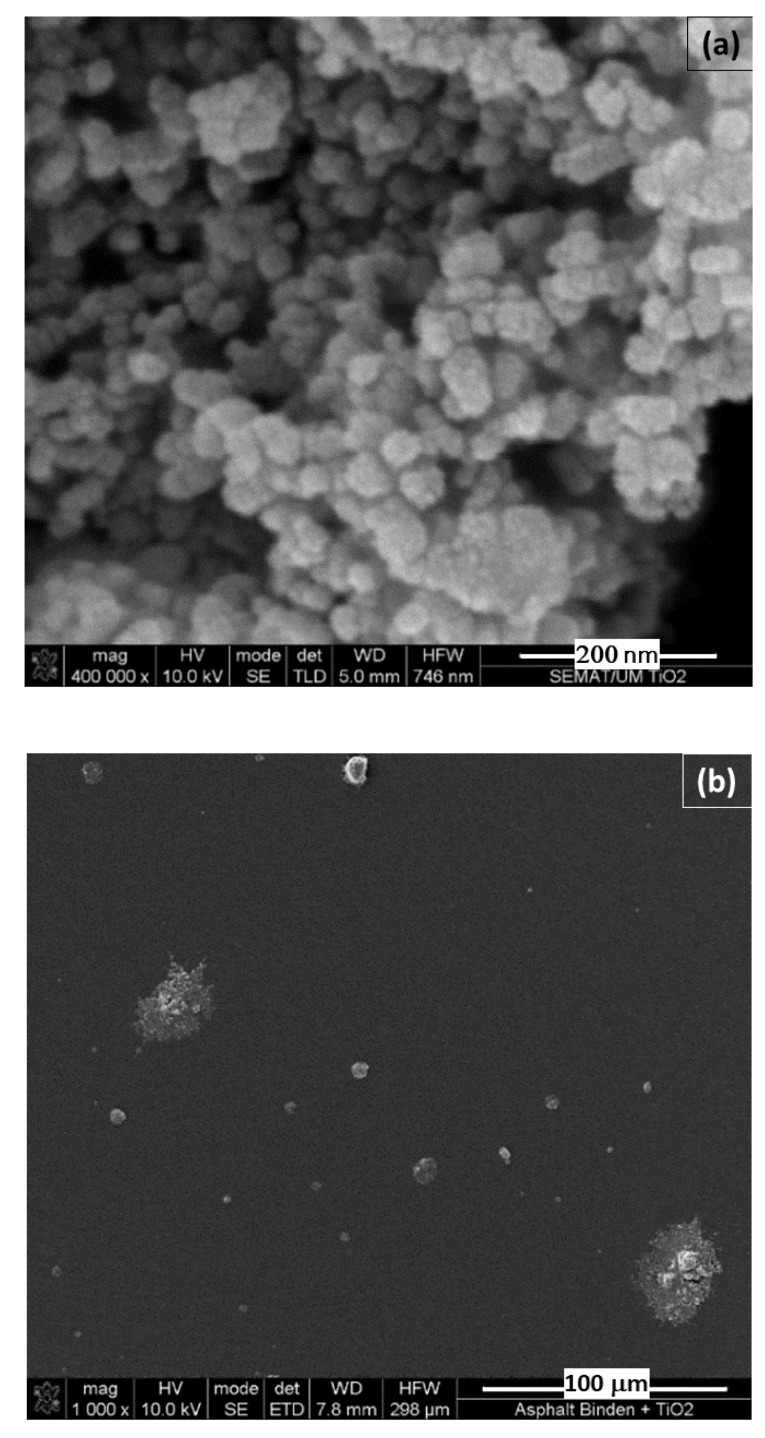
SEM micrograph of the (**a**) powder of TiO_2_ nanoparticles, and (**b**) surface view of a SEM macrograph of asphalt binder modified with nano-TiO_2_.

The asphalt binder was modified with 7 contents of TiO_2_ nanoparticles by mass, i.e., 0.1, 0.25, 0.5, 1, 2, 3, and 6%. Then, it was compared with the reference asphalt binder, denoted as 0%. The modification was carried out with the asphalt binder at 150 °C for 30 min by a low-shear mixer at 1500 RPM. Figure 1 shows scanning electron microscopy (SEM) of the TiO_2_ used in this work and of a real sample of the modified asphalt binder, in which the nanoparticles can be observed.

For the characterization of the material after modification, tests of penetration (ASTM D5), viscosity (ASTM D4402), softening point (ASTM D36), and rheology (by the complex modulus through the DSR—dynamic shear rheometer) (ASTM D7175) were carried out. Then, after short-term aging by the (RTFOT) (ASTM D2872), the mass loss was registered. Moreover, after the RTFOT, the (modified and conventional) asphalt binder was evaluated under penetration, viscosity, softening point, and rheology. The residue aged by RTFOT was subjected to the pressure aging vessel (PAV) aging process (ASTM D6521), being later characterized by the complex modulus and FTIR. The objective of these characterization tests was to evaluate the anti-aging ability of the TiO_2_-modified asphalt binder (in different contents) compared to the reference asphalt binder (0%).

The penetration and softening point tests indicate basic empirical properties of the modified asphalt binders. The mass loss was determined after aging at RTFOT, in which one observes the volatility effects of molecules with low molecular weight. Dynamic viscosity was performed according to the ASTM D4402 standard. The asphalt binder is heated from the lowest to the highest temperature, and viscosity results are acquired at the exact and desired temperatures of 135, 150, 177, and 190 °C. Rheology testing of asphalt binders with nano-TiO_2_ was performed to characterize their viscoelastic behavior. The dynamic shear rheometer (DSR) TA AR 3000 model (Anton Paar, Graz, Austria) was used to determine the two main viscoelastic parameters: complex modulus (G*) and phase angle (δ). The evaluation was performed with temperatures ranging from −10 °C to 90 °C and with 8-mm and 25-mm plate geometries. It was possible to target the identification of rheological data discrepancies through black diagrams (G* × δ). By carrying out this analysis, the behavior of the asphalt binder, the aging effect, and the nanoparticles’ content effect will be better evaluated.

Fourier transform infrared spectroscopy (FTIR) was employed to evaluate the chemical characteristics that result from the modification of the asphalt binder by nano-TiO_2_. The chemical groups of the asphalt binders were analyzed via a Shimadzu IR-Prestige-21 spectrometer (Kyoto, Japan) in a spectral range from 400 cm^−1^ to 4000 cm^−1^. From the FTIR spectra, peak identification was performed, and structural indices of some functional groups (calculated by dividing the peak area of the band under study by the total area of the FTIR spectrum) were determined [41,42].

The RTFOT aging is intended to simulate short-term aging by applying compressed air (4 L/min) over an asphalt binder film subjected to a high temperature of 163 °C for 75 min. Complementarily, the PAV aims to simulate the long-term aging of the asphalt binder. The binder is subjected to 100 °C and a pressure of 2.1 MPa for 22 h.

**Figure 2:**
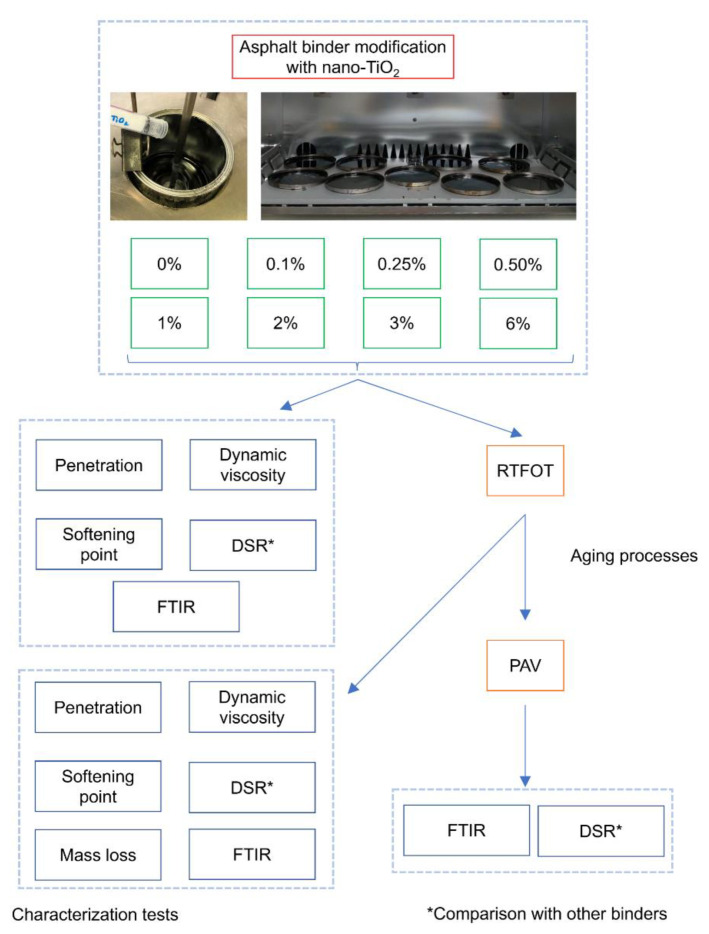
Schematic representation of this research.

Figure 2 corresponds to a schematic representation of the adopted methodology.

## 3. Results and Discussion

Table 1 shows the obtained laboratory results (penetration, softening point, and mass loss), explored later. All the other results (viscosity, DSR, and FTIR) will be presented and discussed in this section.

### 3.1. Penetration

Table 1 shows the results of the penetration tests before and after aging through the RTFOT. After the modification, the asphalt binders with nano-TiO_2_ showed similar penetration values (around 39 × 10^−1^ mm) but lower than the reference binder, 0% (40 × 10^−1^ mm), thus being all classified as asphalt binders 35/50. Increasing the percentage of nano-TiO_2_ leads to a decrease in penetration (before RTFOT), with the greatest reduction occurring for the asphalt binder with the highest percentage modification, 6%.

In general, after the RTFOT, all the asphalt binders decreased their penetration, as expected, due to the aging and loss of lightweight compounds. The different percentages of the modified asphalt binder (after RTFOT) increased the penetration, compared to the reference (0%), except 3% and 6%.

Generally, comparing before and after aging conditions and for the same nanoparticle contents in the asphalt binder, it is observed that the penetration decrease is higher for samples with the highest contents of TiO_2_ (55% and 45% for 3% and 6%, respectively). The penetration of the reference asphalt binder (0%) decreased by 34%. The asphalt binders with 0.1 to 2% presented the lower decrease in this parameter, 28% on average. Therefore, the best asphalt binders were 0.25 and 0.5%, with a penetration decrease of only 24%. The graph concerning the penetration results before and after the RTFOT is properly identified in the Supplementary Materials (SM) in Figure S1.

### 3.2. Softening Point

The softening point results before and after aging by RTFOT are presented in Table 1. When compared to the reference, the asphalt binder modified with nano-TiO_2_ showed a gradual increase in the softening point up to 14%. An inverse behavior is observed after the RTFOT, i.e., the softening point of the modified asphalt binders showed a decrease of up to 9%.

Overall, comparing before and after aging through the RTFOT for the same nanoparticle contents, the reference asphalt binder (0%) increased the softening point up to 31%. The modified binders increased it up to 11%, meaning a reduction in the increase of the softening point with the modification process. The best samples for this analysis were 0.1 and 0.25% (with an increase of 11 and 10%, respectively). Figure S2 plots the softening point results before and after the RTFOT test.

Based on the results of penetration and softening point, it can be concluded that the modified asphalt binders show better properties before the aging process as the softening point increases. After short-term aging (RTFOT), the modification led to an anti-aging contribution, decreasing the softening point when compared to the aged reference binder and reducing the increase in the softening point regarding the same situation before and after the aging process.

### 3.3. Mass Loss

Table 1 also shows the results of the mass loss after the RTFOT test. It is possible to observe a decrease in the mass loss with the asphalt binder modification by nano-TiO_2_. In general, with an increasing percentage of nano-TiO_2,_ there is a gradual and inversely proportional decrease in mass loss. However, an exception occurs for the 1% sample, which could be explained by an unidentified experimental inconsistency. Thus, by increasing the nano-TiO_2_ content, the mass loss was reduced (see Figure S3). It can also be concluded that the incorporation of the semiconductor nanoparticles has an anti-aging effect during the mixing and paving processes of asphalt mixtures (short-term aging).

### 3.4. Dynamic Viscosity

Table 2 presents the dynamic viscosity results before and after aging by RTFOT, which can be graphically analyzed in Figure S4. Before the aging process, most asphalt binders with different nanoparticle contents showed viscosities similar to that obtained for the reference asphalt binder (0%), except 3 and 6%, which displayed higher dynamic viscosity values. For each considered temperature, the average of the percentage changes in the viscosity of the modified binders compared to the reference (0%) is only 6% (for nano-TiO_2_ contents up to 2%). The binders 3 and 6% showed a variation of 39%. After the RTFOT, up to a temperature of 177 °C, the modified asphalt binder with 0.25% nano-TiO_2_ showed a lower viscosity (up to 10%) than the reference asphalt binder (0%). The comparison of the samples for the condition before and after the RTFOT and with the same content of TiO_2_ nanoparticles reveals that the values for the reference asphalt binder increased the dynamic viscosity, on average, by 70%, while the modified ones increased 65%. The lowest increase was for 3% and 0.25% with 40% and 58%, respectively. It can be concluded that, at high temperatures, corresponding to the temperatures of mixing and compaction, nano-TiO_2_ also demonstrated anti-aging capacity.

### 3.5. Rheological Behavior: Complex Modulus and Black Diagram

Table 3 shows the results of the complex modulus before and after the RTFOT aging and after the PAV aging, and Figure S5 plots the corresponding results for the reference asphalt binder, as well as all the modification concentrations versus the reference asphalt binder. Before the RTFOT, the asphalt binders showed similar complex modulus values. At low temperatures (lower than 30 °C), the modified asphalt binders 0.25, 2, and 1% showed a lower complex modulus than the reference asphalt binder (0%). In contrast, the modified asphalt binders 6 and 0.1% showed higher values, and the others presented almost the same complex moduli when compared to the reference. At higher temperatures, the asphalt binders performed similarly.

After the RTFOT, at low temperatures, the binders had similar rheological behavior. At higher temperatures, the modified asphalt binders 0.1, 0.25, 0.5, and 2% presented lower complex moduli than the asphalt binder without TiO_2_ nanoparticles.

After aging by PAV, only performed at high temperatures, the modified binders have lower complex moduli when compared to the reference binder (0%), except for the 3% and 6% modified asphalt binders. This test method could not be performed at low temperatures due to the high stiffness of the binder, due to limitations of the plate geometries, and the used rheometer (for temperatures below 40 °C, asphalt binder samples unstick from the 8 mm plates). Therefore, from the performance of this rheological property, it can be inferred that the modification of the asphalt binder by nano-TiO_2_ ensures an anti-aging effect.

Considering the different contents of nano-TiO_2_ in the asphalt binder samples, the results for most of the previous tests showed non-linear trends regarding anti-aging behavior. Although the insertion of nano-TiO_2_ demonstrates positive effects for the anti-aging properties, the samples with TiO_2_ nanoparticle contents above 2% did not show satisfactory properties, indicating that concentrations above this content are not desired.

**Figure 3:**
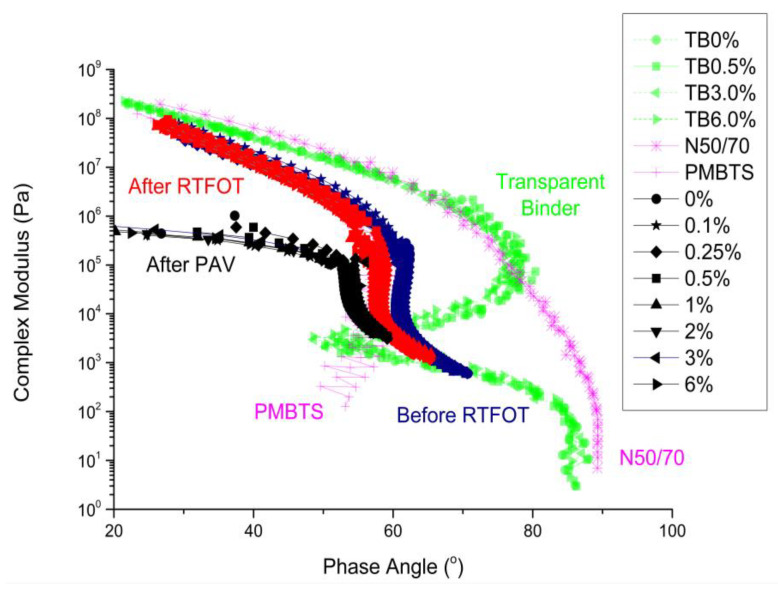
Black diagram for the binders studied in this research versus those from Ref. [43].

In order to better compare the studied asphalt binders, the modified asphalt binders were compared to previously studied asphalt binders, namely: transparent asphalt binder modified with different nano-TiO_2_ contents (coded as TB following the modification content, i.e., 0, 0.5, 3.0, and 6.0%), a conventional asphalt binder (N50/70), and a polymer-modified asphalt binder (PMBTS) from a previous study [43]. For this purpose, the black diagram of all the asphalt binders is presented in Figure 3. As discussed in Ref. [43], N/5070 shows a conventional behavior at the black diagram, smooth, and, while the phase angle increases, the complex modulus decreases. With the increase in the elastic behavior, the curves are shifted to the left (lower phase angles), which occurred for PMBTS and the studied asphalt binders, Elaster with nano-TiO_2_ (0% to 6%). Moreover, before and after the RTFOT, the asphalt binders herein investigated performed similarly to PMBTS at low phase angles (<60 °).

PMBTS presents a plateau near 60° (at a temperature of 58 °C), indicating that the polymer phase presents a continuous elastic network dissolved in a binder with a polymer-dominant phase. However, the asphalt binders herein investigated started performing similarly to the transparent binder for higher phase angles (>60°).

The transparent asphalt binders presented three different regions: (i) from 10^8^ to 5 × 10^5^ Pa—complex modules decrease with increasing phase angles; (ii) from 5 × 10^5^ to 5 × 10^4^ Pa—complex modules decrease with decreasing phase angles; and (iii) below 5 × 10^4^ Pa—the same pattern of the first region. This behavior represents an interchange in the preponderance of the asphalt binder components. This behavior also occurs for Elaster binder. These discontinuities are present in highly asphaltene-structured binder, high wax content bitumen, and highly polymeric binders.

As much as the asphalt binders are aged, they are shifted to the more elastic region (lower phase angles) and, consequently, become more structured binders [44,45]. After the PAV, the complex modulus behavior of the asphalt binders seems not to be affected by different phase angles, losing its phase angle variation sensitivity when compared to the situations before and after the RTFOT. In addition, with increasing aging, the binders present a more solid performance (related to the elastic response) and, consequently, less fluid (related to the viscous response).

### 3.6. FTIR

**Figure 4:**
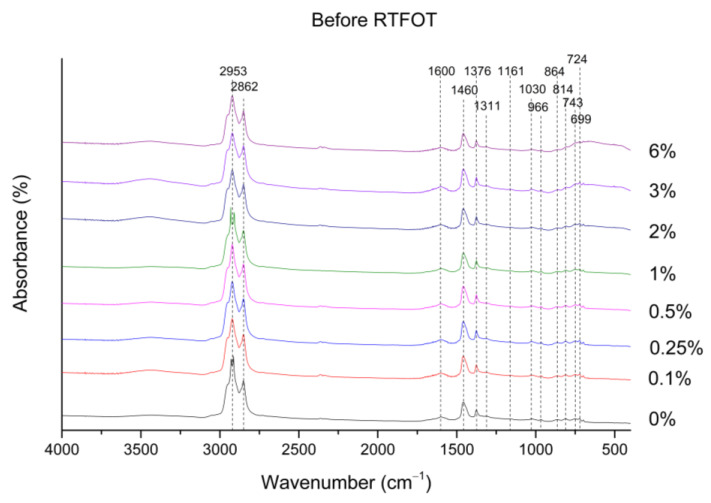
FTIR spectra for all unaged binders investigated in this research work.

The FTIR spectrum of the reference asphalt binder (0%) is compared with the spectra of the asphalt binders modified with nano-TiO_2_. As can be observed in Figure 4, all the unaged asphalt binders revealed similar FTIR spectra, suggesting they have the same chemical structures. In addition, it is noted that the FTIR spectra of the investigated samples are dominated by the strong bands attributed to the asphalt binder. Therefore, it was not possible to identify the Ti–O bond (near to 670 cm^−1^) [46] for the asphalt binders modified with nano-TiO_2_. Nevertheless, the bands at 2953 cm^−1^ and 2862 cm^−1^ are characteristic, respectively, of asymmetric and symmetrical C–H stretching in aliphatic chains [43]. The vibrations at 2862 and 2953 cm^−1^ are known as fundamental vibrations, which arise from the excitation from the ground state to the lowest-energy excited state. Regarding the vibrations at 2953 cm^−1^, it is observed that the 0% spectrum presents a similar peak shape to the 1% spectrum, with a lower resolution. This phenomenon does not result from the presence of another chemical bond, simply due to challenges in the resolution of the spectra obtained by the FTIR technique [47]. Actually, if a fundamental vibration couples with an overtone (integral multiples of the fundamental frequency) or a combination band (sum of the two Interacting bands with different frequencies), the coupled vibration is called a Fermi resonance. This phenomenon is often observed in complex matrices, such as asphalt binders, which have many carbonyl compounds in their composition. The band that appears at 1600 cm^−1^ is associated with C=C stretching vibrations in aromatic rings. The bands near 1460 and 1376 cm^−1^ can be attributed to the asymmetric and symmetrical bending vibrations, respectively, of methyl groups [48], which, in general, are used as a reference because it is anticipated that these aliphatic structures are stable and not affected by the applied aging procedures [49]. The asymmetric and symmetric stretching bands of the S=O (sulfones) appear at 1311 and 1161 cm^−1^, respectively [50]. The band centered at around 1030 cm^−1^ is usually ascribed to the stretching of the S=O_2_ bonds (sulfoxides). Furthermore, this band is directly related to asphalt binder aging [51]. The band appearing at 966 cm^−1^ may correspond to the C–H bending of trans-alkene [52]. The small bands within the 699–864 cm^−1^ interval are attributed to the C–H vibration of benzene [53]. Specifically, the pair 864 cm^−1^ and 814 cm^−1^ can match 1,4-disubstituted rings; the single band at 743 cm^−1^ may be associated with 1,2-disubstituted rings; the long-chain band attributed to the rocking motion of -CH_2_ groups in aliphatic chains can be observed at 724 cm^−1^ [43]. Lastly, the band that appears at 699 cm^−1^ is associated with the out-of-plane bending of the C–H group in monosubstituted aromatic rings [52].

**Figure 5:**
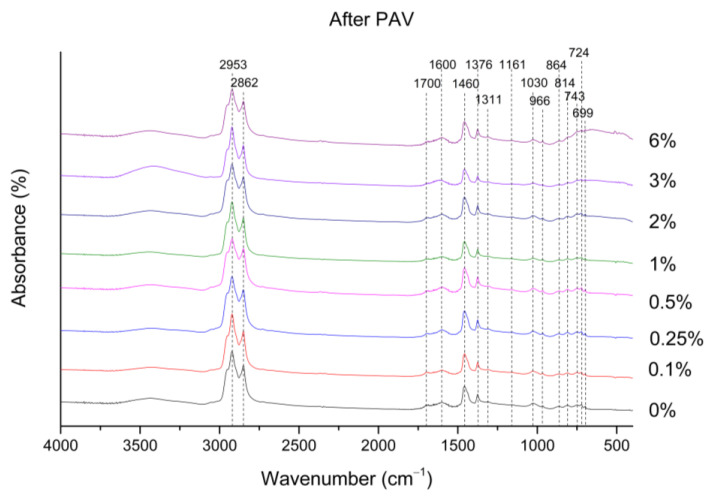
FTIR spectra of RTFOT + PAV aged binders.

**Figure 6:**
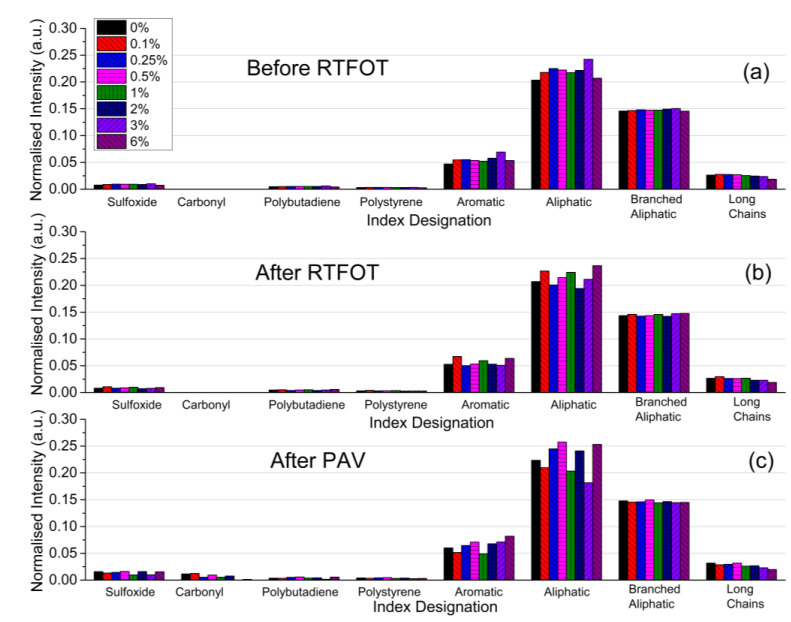
FTIR indices of (**a**) unaged binders, (**b**) RTFOT aged binders, and (**c**) RTFOT + PAV aged binders.

The trends in the FTIR spectra of the RTFOT aged binder (data not shown) are similar to those in Figure 4 (unaged binders). For the case of the RTFOT+PAV aged binder, a new band at 1700 cm^−1^ was observed, which can be attributed to the carbonyl group (see Figure 5). In addition, a slight increase in the number of sulfoxides (1030 cm^−1^) is observed after the PAV, as shown in Figure 6. According to the literature, the increase in the sulfoxide (possibly due to the increase in the asphaltenes content) and carbonyl bands (generally due to the formation of ketones, dicarboxylic anhydrides, and carboxylic acids) is linked to the aging mechanism of the asphalt binder [54].

## 4. Conclusions

This article is devoted to evaluating the anti-aging effect of TiO_2_ in an asphalt binder after being subjected to standard aging processes, namely RTFOT for the short-term effect and PAV for the long-term effect. A commercial binder was modified by nano-TiO_2_ using seven different percentages and subsequently subjected to physical and rheological tests to identify the potential effects of these nanoparticle contents on the anti-aging properties. The following conclusions can be reported:

- After modification and before the aging process, the nano-TiO_2_-modified binder decreased the penetration value when compared to the reference binder. After the RTFOT, the modified binder increased the penetration when compared to the reference, except for the samples with higher TiO_2_ contents (3% and 6%). Regarding the softening point, before aging, the addition of nano-TiO_2_ led to an increase in this parameter. After the RTFOT, it led to an attenuation of the softening point increase with the modification process. The increase in the TiO_2_ percentage led to a gradual decrease in mass loss. These tests showed that the nano-TiO_2_-modified binders positively effect short-term aging.
- For the dynamic viscosity, there was evidence of an anti-aging effect regarding the asphalt behavior at high temperatures (similar to asphalt mixtures mixing and compaction temperatures).
- Before the RTFOT, the modified asphalt binders maintained the complex modulus value regarding the results of the reference binder. After the RTFOT, lower TiO_2_ percentages (0.1, 0.25, 0.5, and 2%) showed a lower complex modulus than the reference binder at higher temperatures. After the PAV, the modified binders had lower complex moduli when compared to the reference binder, except 3 and 6%.
- The asphalt binder investigated presented an alternation of the behavior regarding the black diagram. With the aging, as much as the asphalt binders are aged, they are shifted to the more elastic region (lower phase angles) and, consequently, are more structured binders, performing more solid and, consequently, less fluid.
- Regarding the FTIR presented only after the PAV, there was a decreasing trend of the carbonyl band with increasing TiO_2_ content. Moreover, a slight increase in the number of sulfoxides was observed after the PAV when compared to the results before and after the RTFOT.
- The evaluation of the properties related to aging indicated satisfactory results for percentages of modification below 2%. However, the 0.25% nano-TiO_2_ content accumulated the greatest number of best testing results, which indicates this concentration has the greatest anti-aging potential.

Briefly, nano-TiO_2_ showed an anti-aging effect in the investigated asphalt binder, which can mitigate problems caused by increased stiffness. In general, from the results presented above, it is observed that, after aging, TiO_2_ prevents the high increase in the stiffness of the asphalt binder, which is corroborated by the penetration and softening point results. On the other hand, after the RTFOT, TiO_2_ showed an anti-aging contribution, corroborated by the penetration, softening point, and mass loss tests. Moreover, after the PAV, the long-term anti-aging effect was corroborated by the presence of the carbonyl group and complex moduli decrease. However, the tests showed a non-linear trend regarding the anti-aging result for the different nano-TiO_2_ contents. The samples with nanoparticle contents above 2% did not show satisfactory properties regarding the anti-aging process, thus indicating that content above 2% is not desired. Therefore, incorporating TiO_2_ nanoparticles into asphalt mixtures would provide an environmental and sustainable gain, extending the lifetimes of asphalt pavement roads. The future phase of this research work will deal with aging carried out inside a UV light chamber; the objective is to analyze the aging phenomenon after the material has been irradiated with UV light to consolidate the modification of the asphalt binder using nano-TiO_2_ for anti-aging effects.

## Figures and Tables

**Table 1:** Results for penetration, softening point, and mass loss.

TiO_2_ Content (%)	Penetration (10^−1^ mm)		Softening Point (°C)		Mass Loss (%)
	Before RTFOT	After RTFOT	Before RTFOT	After RTFOT	
0	40	27	65	85	0.57
0.1	39	28	73	81	0.57
0.25	39	29	71	78	0.53
0.50	39	29	71	77	0.50
1	39	27	73	79	0.38
2	39	27	72	78	0.48
3	40	18	73	79	0.48
6	37	20	74	81	0.45

**Table 2:** Results of dynamic viscosity at different temperatures 135, 150, 177, and 190 °C.

TiO_2_ Content (%)	Dynamic Viscosity (cP)							
	Before RTFOT (135 °C)	After RTFOT (135 °C)	Before RTFOT (150 °C)	After RTFOT (150 °C)	Before RTFOT (177 °C)	After RTFOT (177 °C)	Before RTFOT (190 °C)	After RTFOT (190 °C)
0	2784	5588	1138	2163	388	600	271	363
0.1	2909	5958	1209	2383	409	638	263	400
0.25	2638	5038	1175	1988	442	592	271	375
0.5	2875	5729	1204	2250	413	625	263	413
1	2825	5625	1275	2242	438	650	288	400
2	3117	6496	1363	2384	450	650	279	413
3	4463	7458.	1400	2150	500	613	334	388
6	4058	8429	1663	3121	554	879	388	554

**Table 3:** (**a**) Results of complex modulus for the modified asphalt binders 0%, 0.1%, and 0.25%. (**b**) Results of complex modulus for the modified asphalt binders 0.5%, 1%, and 2%. (**c**) Results of complex modulus for the modified asphalt binders 3% and 6%.

**(a)**									
**Temperature (°C)**	**Complex Modulus (Pa)**								
	**Before RTFOT**	**After RTFOT**	**After PAV**	**Before RTFOT**	**After RTFOT**	**After PAV**	**Before RTFOT**	**After RTFOT**	**After PAV**
	**TiO_2_ Content (%)**								
	**0**			**0.1**			**0.25**		
0	2.61 × 10^7^	3.90 × 10^7^		4.28 × 10^7^	6.29 × 10^7^		1.87 × 10^7^	4.54 × 10^7^	
10	6.99 × 10^6^	1.12 × 10^7^		1.07 × 10^7^	1.89 × 10^7^		5.02 × 10^6^	1.37 × 10^7^	
20	1.38 × 10^6^	2.76 × 10^6^		2.17 × 10^6^	4.47 × 10^6^		1.05 × 10^6^	3.28 × 10^6^	
30	2.81 × 10^5^	6.62 × 10^5^		4.51 × 10^5^	6.94 × 10^5^		2.22 × 10^5^	7.62 × 10^5^	
40	7.82 × 10^4^	1.68 × 10^5^	5.69 × 10^5^	7.39 × 10^4^	1.74 × 10^5^	5.08 × 10^5^	7.19 × 10^4^	1.32 × 10^5^	4.69 × 10^5^
50	2.28 × 10^4^	5.30 × 10^4^	1.69 × 10^5^	2.33 × 10^4^	5.18 × 10^4^	1.51 × 10^5^	2.18 × 10^4^	3.99 × 10^4^	1.29 × 10^5^
60	8.09 × 10^3^	1.86 × 10^4^	5.70 × 10^4^	8.72 × 10^3^	1.78 × 10^4^	4.97 × 10^4^	7.72 × 10^3^	1.42 × 10^4^	4.25 × 10^4^
70	3.21 × 10^3^	7.47 × 10^3^	2.24 × 10^4^	3.65 × 10^3^	7.07 × 10^3^	2.08 × 10^4^	3.13 × 10^3^	5.75 × 10^3^	1.63 × 10^4^
80	1.37 × 10^3^	3.28 × 10^3^	9.66 × 10^3^	1.64 × 10^3^	3.11 × 10^3^	8.41 × 10^3^	1.38 × 10^3^	2.56 × 10^3^	6.99 × 10^3^
90	6.11 × 10^2^	1.50 × 10^3^	4.37 × 10^3^	7.67 × 10^2^	1.41 × 10^3^	3.87 × 10^3^	6.21 × 10^2^	1.18 × 10^3^	3.19 × 10^3^
**(b)**									
**Temperature (°C)**	**Complex Modulus (Pa)**								
	**Before RTFOT**	**After RTFOT**	**After PAV**	**Before RTFOT**	**After RTFOT**	**After PAV**	**Before RTFOT**	**After RTFOT**	**After PAV**
	**TiO_2_ Content (%)**								
	**0.5**			**1**			**2**		
0	2.60 × 10^7^	5.15 × 10^7^		2.18 × 10^7^	3.70 × 10^7^		2.21 × 10^7^	4.99 × 10^7^	
10	6.53 × 10^6^	1.47 × 10^7^		5.63 × 10^6^	1.13 × 10^7^		5.58 × 10^6^	1.41 × 10^7^	
20	1.33 × 10^6^	3.41 × 10^6^		1.16 × 10^6^	2.82 × 10^6^		1.14 × 10^6^	3.41 × 10^6^	
30	2.75 × 10^5^	5.00 × 10^5^		2.43 × 10^5^	6.89 × 10^5^		6.64 × 10^5^	4.39 × 10^5^	
40	7.52 × 10^4^	1.51 × 10^5^	4.85 × 10^5^	7.48 × 10^4^	2.10 × 10^5^	6.13 × 10^5^	7.71 × 10^4^	1.64 × 10^5^	5.43 × 10^5^
50	2.27 × 10^4^	4.49 × 10^4^	1.38 × 10^5^	2.23 × 10^4^	6.06 × 10^4^	1.75 × 10^5^	2.40 × 10^4^	4.98 × 10^4^	1.58 × 10^5^
60	7.91 × 10^3^	1.56 × 10^4^	4.61 × 10^4^	7.95 × 10^3^	2.04 × 10^4^	5.44 × 10^4^	8.66 × 10^3^	1.79 × 10^4^	5.07 × 10^4^
70	3.19 × 10^3^	6.24 × 10^3^	1.79 × 10^4^	3.28 × 10^3^	8.03 × 10^3^	2.04 × 10^4^	3.54 × 10^3^	7.30 × 10^3^	1.94 × 10^4^
80	1.38 × 10^3^	2.74 × 10^3^	7.60 × 10^3^	1.44 × 10^3^	3.44 × 10^3^	8.55 × 10^3^	1.56 × 10^3^	3.24 × 10^3^	8.29 × 10^3^
90	6.14 × 10^2^	1.25 × 10^3^	3.44 × 10^3^	6.53 × 10^2^	1.53 × 10^3^	3.85 × 10^3^	7.13 × 10^2^	1.49 × 10^3^	3.76 × 10^3^
**(c)**									
**Temperature (°C)**				**Complex Modulus (Pa)**					
				**Before RTFOT**	**After RTFOT**	**After PAV**	**Before RTFOT**	**After RTFOT**	**After PAV**
				**TiO_2_ Content (%)**					
				**3**			**6**		
0				2.70 × 10^7^	4.68 × 10^7^		3.16 × 10^7^	4.09 × 10^7^	
10				7.31 × 10^6^	1.38 × 10^7^		8.30 × 10^6^	1.28 × 10^7^	
20				1.56 × 10^6^	3.35 × 10^6^		1.75 × 10^6^	3.27 × 10^6^	
30				3.38 × 10^5^	8.08 × 10^5^		3.76 × 10^5^	7.66 × 10^5^	
40				8.61 × 10^4^	1.89 × 10^5^	6.83 × 10^5^	7.85 × 10^4^	2.54 × 10^5^	6.28 × 10^5^
50				2.52 × 10^4^	5.62 × 10^4^	1.92 × 10^5^	2.48 × 10^4^	7.34 × 10^4^	2.17 × 10^5^
60				8.91 × 10^3^	1.98 × 10^4^	6.19 × 10^4^	9.19 × 10^3^	2.52 × 10^4^	6.86 × 10^4^
70				3.63 × 10^3^	7.95 × 10^3^	2.36 × 10^4^	3.88 × 10^3^	9.99 × 10^3^	2.61 × 10^4^
80				1.58 × 10^3^	3.52 × 10^3^	1.00 × 10^4^	1.79 × 10^3^	4.39 × 10^3^	1.11 × 10^4^
90				7.17 × 10^2^	1.62 × 10^3^	4.50 × 10^3^	8.55 × 10^2^	2.02 × 10^3^	4.78 × 10^3^

## Data Availability

Not applicable.

## Supplementary Materials

The following supporting information can be downloaded at: https://www.mdpi.com/article/10.3390/nano12101678/s1, Figure S1: Penetration results: (**a**) before, and (**b**) after RTFOT; Figure S2: Softening Point results: (**a**) before RTFOT, and (**b**) after RTFOT; Figure S3: Results of Mass Loss after RTFOT; Figure S4: Results of Dynamic Viscosity: (**a**) before RTFOT, and (**b**) after RFTOT; Figure S5: Complex Moduli results before RTFOT, after RTFOT, and after PAV for: (**a**) reference asphalt binder (0%); (**b**) 0% versus 0.1%; (**c**) 0% versus 0.25%; (**d**) 0% versus 0.5%; (**e**) 0% versus 1%; (**f**) 0% versus 2%; (**g**) 0% versus 3%, and (**h**) 0% versus 6%.
