# Supplementary material for: Asphalt Binder “Skincare”? Aging Evaluation of an Asphalt Binder Modified by Nano-TiO2
Source: Nanomaterials (Basel). 2022 May 14;12(10):1678. doi: 10.3390/nano12101678 (PMC9145936; doi:10.3390/nano12101678)
Supplement: Supplementary file 1 [file nanomaterials-12-01678-s001.zip › nanomaterials-1641563-supplementary.pdf]

Supplementary Materials

Asphalt Binder "Skincare"? Aging Evaluation of an Asphalt Binder Modified by Nano-TiO_2

Orlando Lima Jr. [1,†], Cátia Afonso [2,†], Iran Rocha Segundo [1,2,*], Salmon Landi Jr. [3], Natália C. Homem [4], Elisabete Freitas [1], Amanda Alcantara [5], Verônica Castelo Branco [5], Sandra Soares [5], Jorge Soares [5], Vasco Teixeira [2,*] and Joaquim Carneiro [2,*]

[1] Department of Civil Engineering, Institute for Sustainability and Innovation in Structural Engineering (ISISE), University of Minho, 4800-058 Guimarães, Portugal; orlandojunior.jr@hotmail.com (O.L.J.); efreitas@civil.uminho.pt (E.F.)
[2] Centre of Physics of Minho and Porto Universities (CF-UM-UP), Azurém Campus, University of Minho, 4800-058 Guimarães, Portugal; catiaj_afonso@hotmail.com
[3] Federal Institute Goiano, Rio Verde 75901-970, Brazil; salmon.landi@ifgoiano.edu.br
[4] Digital Transformation CoLab (DTx), Building 1, Campus of Azurém, University of Minho, 4800-058 Guimarães, Portugal; natalia.homem@dtx-colab.pt
[5] Departamento de Engenharia de Transportes, Universidade Federal do Ceará, Fortaleza 60455-760, Brazil; amanda.a@det.ufc.br (A.A.); veronica@det.ufc.br (V.C.B.); sas@ufc.br (S.S.); jsoares@det.ufc.br (J.S.)
* Correspondence: iran_gomes@hotmail.com (I.R.S.); vasco@fisica.uminho.pt (V.T.); carneiro@fisica.uminho.pt (J.C.)
† These authors contributed equally to this work.

This supplementary information serves to provide further information on the content addressed in the main article. Figures S1, S2, and S3 are related to the laboratory results, namely penetration, softening point, and mass loss presented in Table 1. In order to support the analysis, a trend line was added to the graphical results presented next.

Penetration

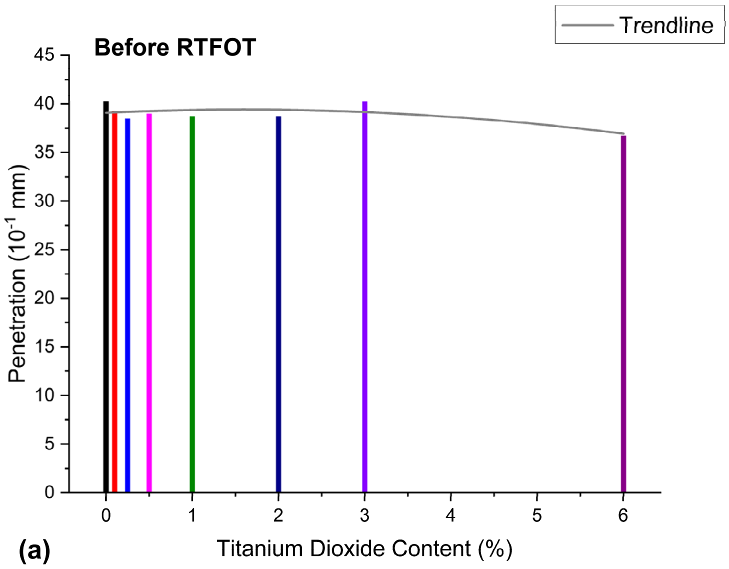

(a)

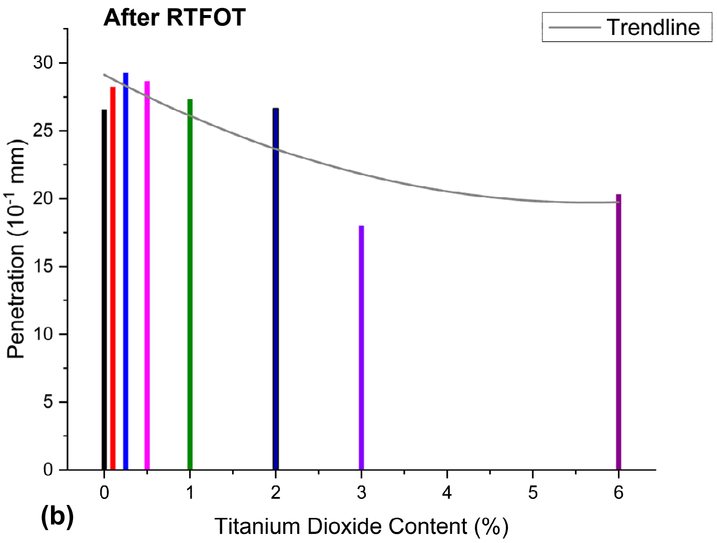

Figure S1. Penetration results: (**a**) before, and (**b**) after RTFOT.

Softening Point

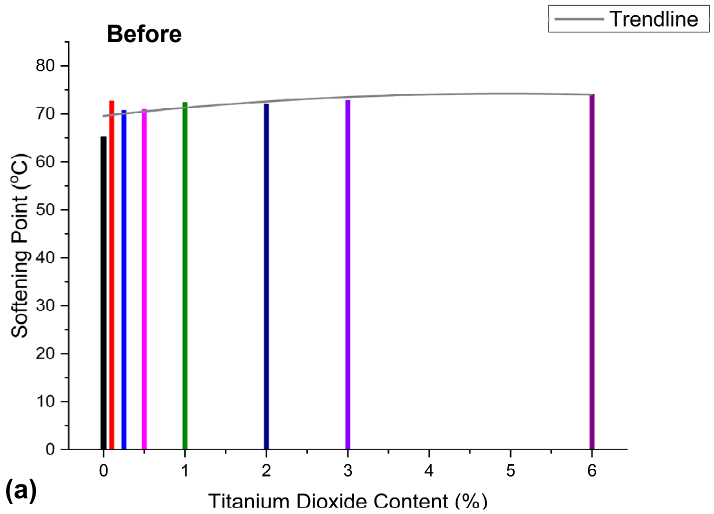

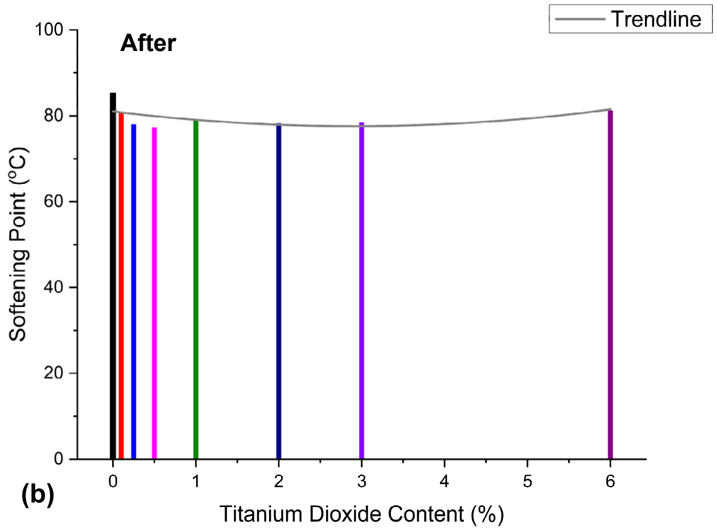

Figure S2. Softening Point results: (**a**) before RTFOT, and (**b**) after RTFOT.

Mass Loss

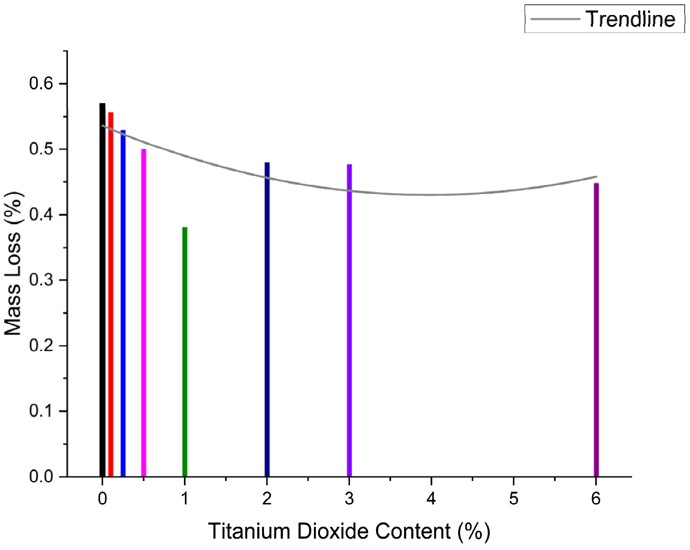

Figure S3. Results of Mass Loss after RTFOT.

Figure S4 concerns the dynamic viscosity results before and after the RTFOT test, which are presented in the main article in Table 2. On the other hand, Figure S5 shows the results about the rheological behavior, namely the complex modulus, which, in the main article, is demonstrated in Table 3. This figure refers to the results for the reference asphalt binder, as well as for all modification concentrations versus the reference asphalt binder, before and after the RTFOT aging and after the PAV aging. These assessments enable to compare the influence of the nano-TiO_2 content on the modification, separately, to ensure that the graphical information does not overlap or become difficult to interpret.

Dynamic Viscosity

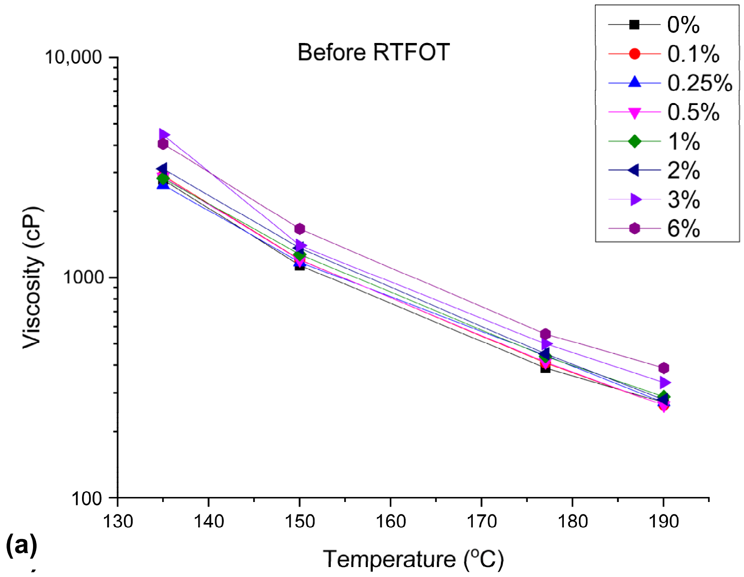

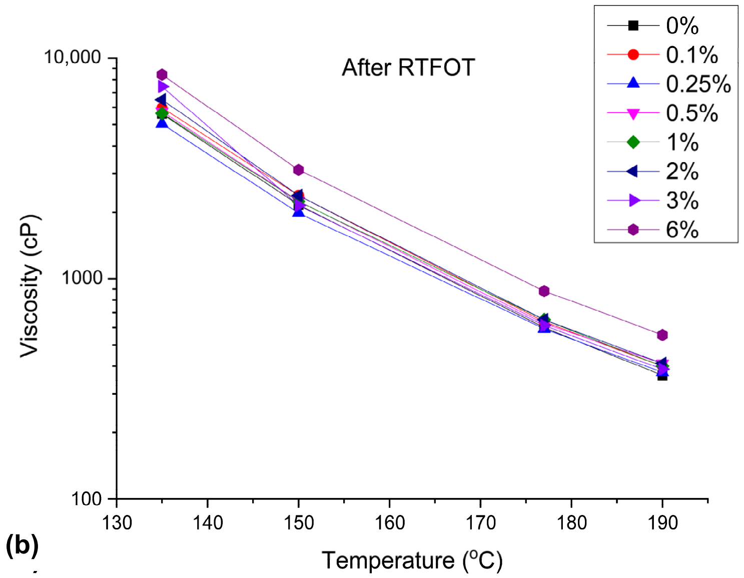

Figure S4. Results of Dynamic Viscosity: (**a**) before RTFOT, and (**b**) after RFTOT.

Rheological Behavior: Complex Modulus

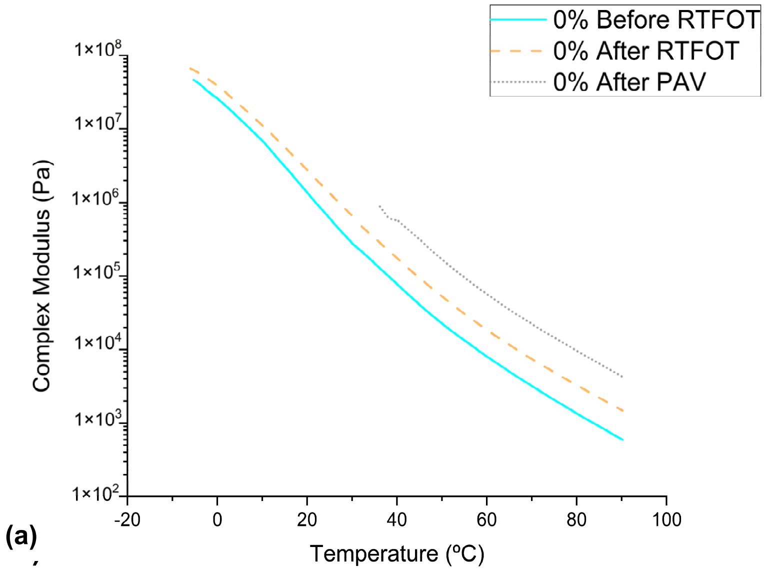

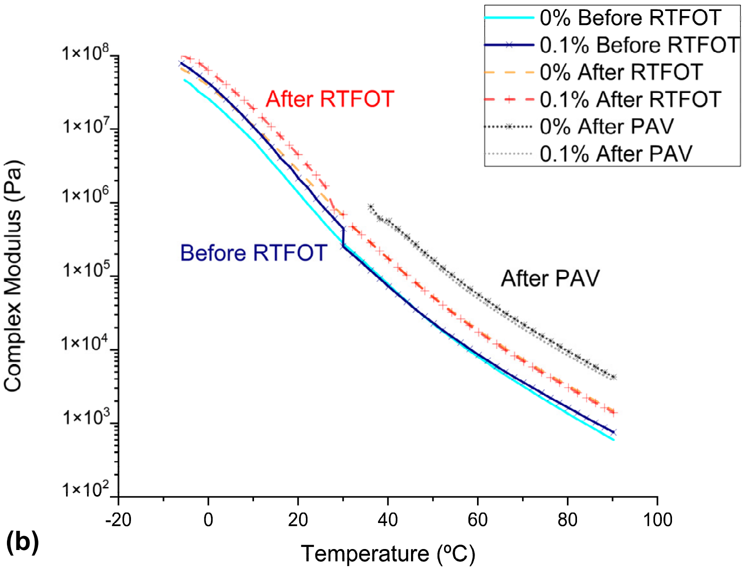
0% Before RTFOT
0.1% Before RTFOT
0% After RTFOT
0.1% After RTFOT
0% After PAV
0.1% After PAV
After RTFOT
Before RTFOT
After PAV
Complex Modulus (Pa)
Temperature (ºC)

(b)

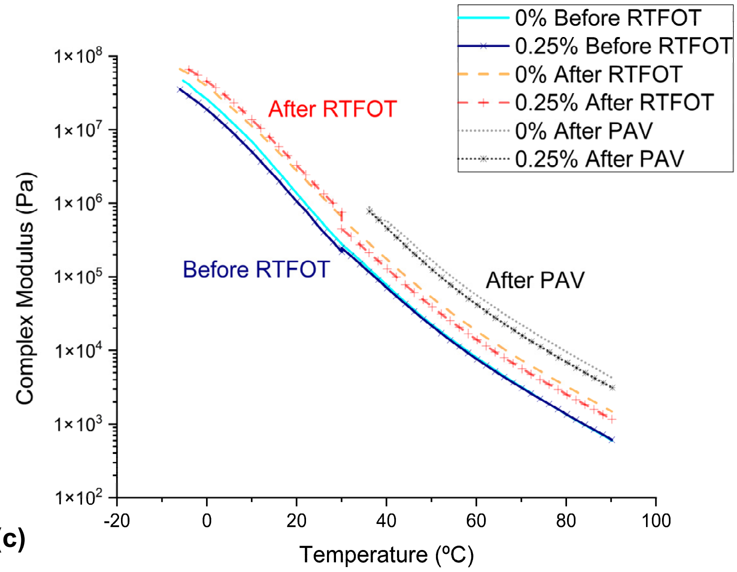
0% Before RTFOT
0.25% Before RTFOT
0% After RTFOT
0.25% After RTFOT
0% After PAV
0.25% After PAV
After RTFOT
Before RTFOT
After PAV
Complex Modulus (Pa)
Temperature (ºC)

(c)

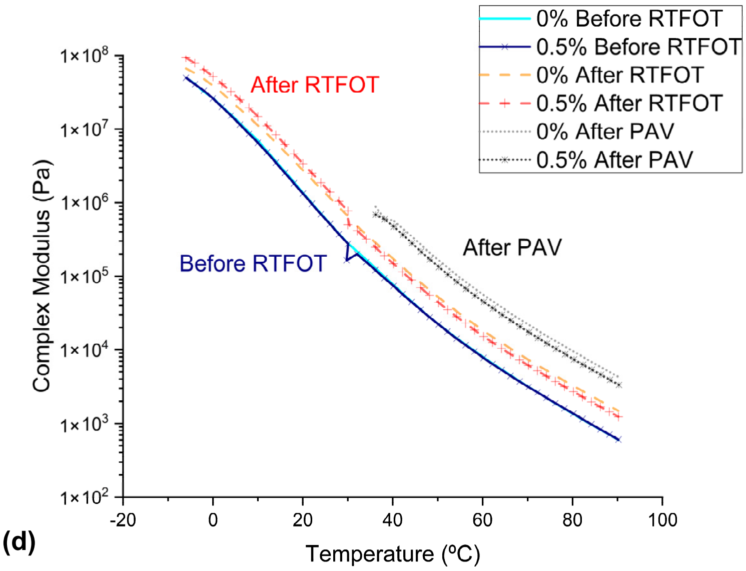
0% Before RTFOT
0.5% Before RTFOT
0% After RTFOT
0.5% After RTFOT
0% After PAV
0.5% After PAV
After RTFOT
Before RTFOT
After PAV
Complex Modulus (Pa)
Temperature (ºC)

(d)

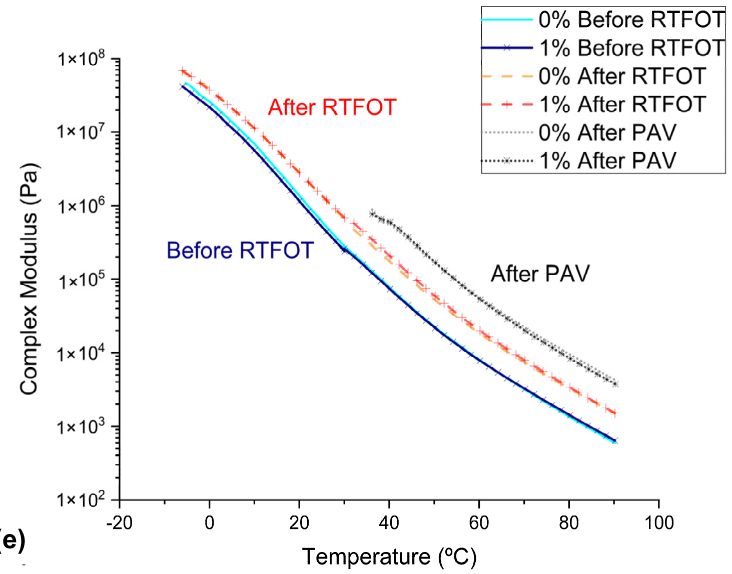
0% Before RTFOT
1% Before RTFOT
0% After RTFOT
1% After RTFOT
0% After PAV
1% After PAV
After RTFOT
Before RTFOT
After PAV
Complex Modulus (Pa)
Temperature (ºC)

(e)

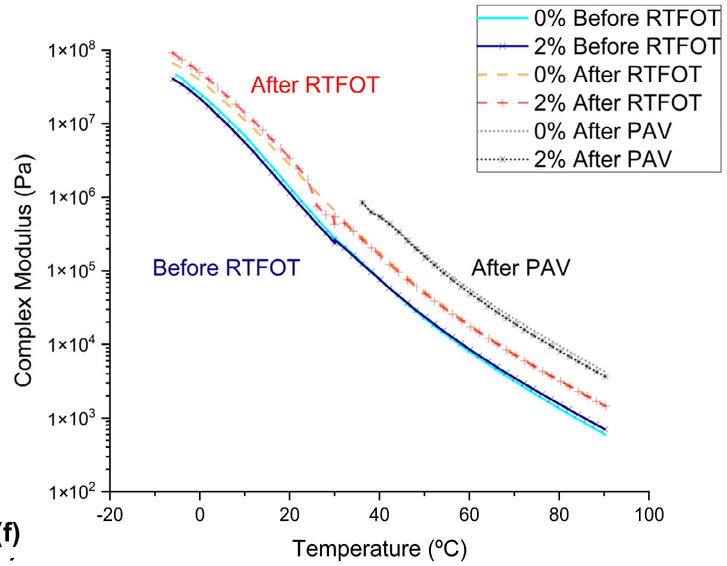
0% Before RTFOT
2% Before RTFOT
0% After RTFOT
2% After RTFOT
0% After PAV
2% After PAV
After RTFOT
Before RTFOT
After PAV
Complex Modulus (Pa)
Temperature (ºC)

(f)

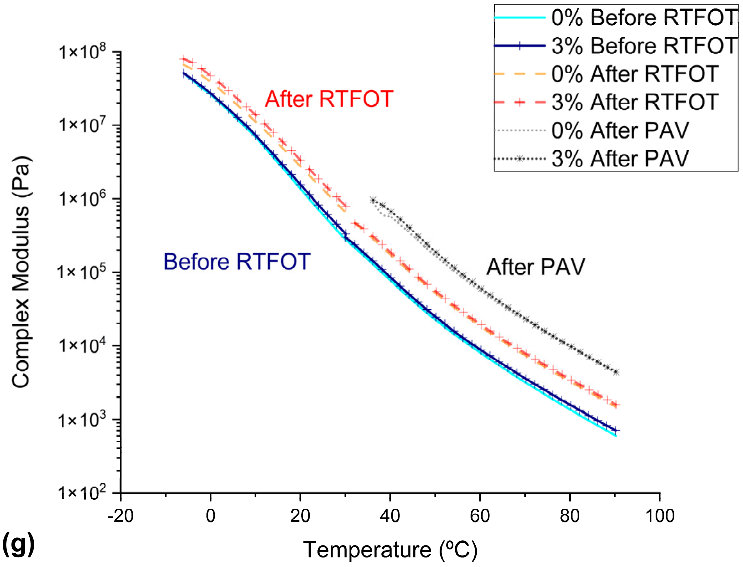
0% Before RTFOT
3% Before RTFOT
0% After RTFOT
3% After RTFOT
0% After PAV
3% After PAV
After RTFOT
Before RTFOT
After PAV
Complex Modulus (Pa)
Temperature (ºC)

(g)

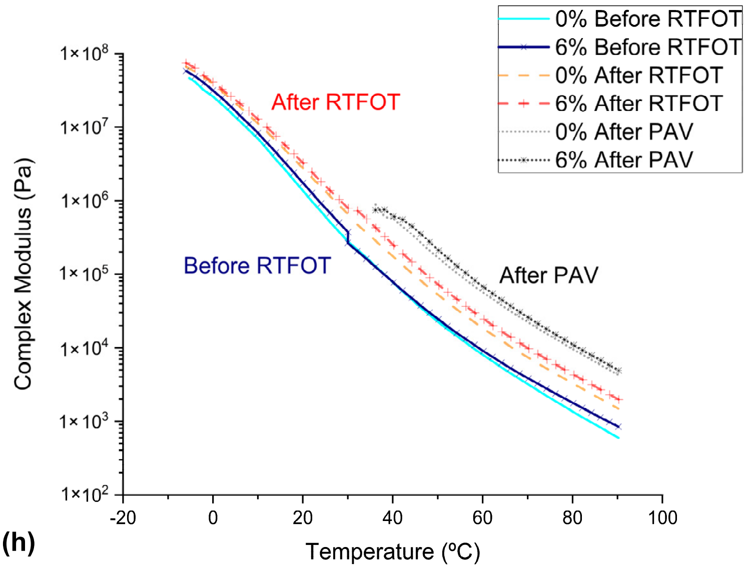

(h)

Figure S5. Complex Moduli results before RTFOT, after RTFOT, and after PAV for: (**a**) reference asphalt binder (0%); (**b**) 0% versus 0.1%; (**c**) 0% versus 0.25%; (**d**) 0% versus 0.5%; (**e**) 0% versus 1%; (**f**) 0% versus 2%; (**g**) 0% versus 3%, and (**h**) 0% versus 6%.
